# The Role of the Laser-Induced Oxide Layer in the Formation of Laser-Induced Periodic Surface Structures

**DOI:** 10.3390/nano10010147

**Published:** 2020-01-14

**Authors:** Camilo Florian, Jean-Luc Déziel, Sabrina V. Kirner, Jan Siegel, Jörn Bonse

**Affiliations:** 1 Bundesanstalt für Materialforschung und -prüfung (B A M), Unter den Eichen 87, 12205 Berlin, Germany; Sabrina.Kirner@gmx.net; 2 Département de Physique, Université Laval, Pavillon Alexandre-Vachon 1045, Av. de la Médecine, Québec, QC G1V0A6, Canada; jean-luc.deziel.1@ulaval.ca; 3 Laser Processing Group, Instituto de Óptica IO-CSIC, Serrano 121, 28006 Madrid, Spain; j.siegel@io.cfmac.csic.es

**Keywords:** laser-induced oxide layer, laser-induced periodic surface structures, LIPSS, surface chemistry, nanostructuring, femtosecond laser processing

## Abstract

Laser-induced periodic surface structures (LIPSS) are often present when processing solid targets with linearly polarized ultrashort laser pulses. The different irradiation parameters to produce them on metals, semiconductors and dielectrics have been studied extensively, identifying suitable regimes to tailor its properties for applications in the fields of optics, medicine, fluidics and tribology, to name a few. One important parameter widely present when exposing the samples to the high intensities provided by these laser pulses in air environment, that generally is not considered, is the formation of a superficial laser-induced oxide layer. In this paper, we fabricate LIPSS on a layer of the oxidation prone hard-coating material chromium nitride in order to investigate the impact of the laser-induced oxide layer on its formation. A variety of complementary surface analytic techniques were employed, revealing morphological, chemical and structural characteristics of well-known high-spatial frequency LIPSS (HSFL) together with a new type of low-spatial frequency LIPSS (LSFL) with an anomalous orientation parallel to the laser polarization. Based on this input, we performed finite-difference time-domain calculations considering a layered system resembling the geometry of the HSFL along with the presence of a laser-induced oxide layer. The simulations support a scenario that the new type of LSFL is formed at the interface between the laser-induced oxide layer and the non-altered material underneath. These findings suggest that LSFL structures parallel to the polarization can be easily induced in materials that are prone to oxidation.

## 1. Introduction

The fabrication of laser-induced periodic surface structures (LIPSS) on metals, semiconductors and dielectrics can be realized with linearly polarized high intensity ultrashort laser pulses [1]. These structures are parallel line-grating-like modifications formed at the sample surface in a self-ordered way either parallel (∥) or perpendicular (⊥) to the laser beam polarization. Their periodicity ($\Lambda_{\mathrm{LIPSS}}$) usually ranges from hundreds of nanometers up to some micrometers and it is used to classify them into the general categories as low-spatial frequency LIPSS (LSFL), when $\Lambda_{\mathrm{LSFL}}\;\sim \;\lambda$, and high-spatial frequency LIPSS (HSFL) for $\Lambda_{\mathrm{HSFL}}\ll \lambda$, where λ is the laser wavelength [2]. Suitable manufacturing strategies have been identified, including the optimization of laser processing parameters (laser fluence, wavelength, repetition rate, angle of incidence, number of pulses per spot area) [3,4,5,6,7,8], material properties (optical, thermal and mechanical properties) [9,10,11], and the ambient medium in which they are generated (air, vacuum, reactive atmospheres) [12,13,14,15] for applications in optics, medicine, fluid transport and tribology among others [1].

The abundance of parameters and the complexity of the mechanisms involved pose difficulties to develop a model that satisfyingly describes experimental findings. However, there have been recent significant advances in models based on finite-difference time-domain calculations (FDTD) that describe the formation of ablative LSFL in order to account for the periodicity of LIPSS on various materials under realistic irradiation conditions, including the formation of random defects at the surface [16]. For metals, Rudenko et al. [17] implemented a numerical FDTD approach for calculating the electromagnetic fields scattered from ensembles of isolated dipole-like scatterers. In addition, FDTD simulations were used for the description of the processes that occur when different types of structures are formed in dielectrics under femtosecond laser irradiation [18]. In the latter work, the formation mechanisms of different types of LIPSS including LSFL parallel and HSFL perpendicular to the polarization were presented consistently with experimental demonstrations and for the LSFL in line with an analytical theory [19]. Based on the intrinsic optical properties of the irradiated materials, FDTD simulations allow to classify the periodic structures into specific categories [16,20,21], including type-d (“dissident”) structures corresponding to LSFL being parallel to the polarization with periodicities $\Lambda_{\mathrm{LSFL}}$ ~ λ*/n* (with *n* being the refractive index of material), type-s (“scattering”) for LSFL perpendicular to the polarization with $\Lambda_{\mathrm{LSFL}}$ ~ λ, type-m (“mixed”) for a special kind of LSFL parallel to the polarization with periodicities $\Lambda_{\mathrm{LSFL}}$ ~ λ, and type-r (“roughness”) for HSFL structures that typically are perpendicular to the laser polarization and $\Lambda_{\mathrm{HSFL}}$ << λ. Fuentes-Edfuf et al. [8] recently published a study on the formation of LIPSS in metals where the key parameters analyzed were the beam incident angle and the role of surface roughness for the generation of surface plasmons that are ultimately responsible for the formation of LIPSS. The experiments were done in air atmosphere at room temperature using fluences above the ablation threshold. Thus, the temperatures reached are high enough to allow the sample oxidation on the irradiated areas. Despite that those studies include experimental results in good agreement with simulations, the laser-induced oxidation was not considered. Usually, this effect is only accounted when the final application for which the LIPSS where fabricated is compromised, such as in the case of surface wetting [22] and tribology [23].

About 20 years ago, the fs-laser ablation behavior of TiN, CrN and other hard-coating materials was studied in detail by several groups using low repetition rate (≤1 kHz) Titanium:Sapphire (Ti:Sa) laser pulses at 790 nm wavelength [24,25]. HSFL with an orientation perpendicular to the laser beam polarization and periods of 100–200 nm were found at fluences close to the ablation threshold [24]. At gradually increasing fluences, the structures showed a “smooth transition” of the HSFL towards LSFL of larger periods featuring the same orientation [25]. These structures are termed ${\mathrm{LSFL}}^{⊥}$ in the following. Large area chemical analyzes revealed that the HSFL were formed in the regime of strong superficial oxidation [24] but an explicit link between the LIPSS topography and surface chemistry was not established. Recently, Kirner et al. presented the first spatially and depth-resolved investigation of both HSFL and ${\mathrm{LSFL}}^{⊥}$ formed upon fs-laser scan processing of titanium surfaces in air environment [26]. These depths-profiling Auger microscopy measurements revealed that a superficial oxide layer is accompanying the formation of LIPSS in the ablative regime. Its extent of more than 150 nm into the bulk largely exceeds the topographical depth modulation of the HSFL, which typically accounts for some tens of nanometers only. However, this “classical regime” of ablative LIPSS formation has to be distinguished from other processing regimes of LIPSS that may manifest in the sub-ablative regime through localized heat-induced effects: Öktem et al. [27] presented a detailed study on the formation of superficial oxide-based periodic structures in metals by inducing the incorporation of oxygen to form oxides (specifically titanium dioxide and tungsten oxide). This process occurs when the incoming focused laser beam interferes with radiation scattered at existing nanostructures or surface defects leading to the imprint of a periodic fluence pattern of maxima and minima, allowing the incorporation of oxygen in the places where the intensity maxima is located [27]. Dostovalov et al. [28,29] very recently published several works on thermochemical formation of elevated parallel and perpendicular surface structures on metallic chromium films produced by local oxidation at the local intensity maxima produced by high-repetition rate (200 kHz) laser pulses in the non-ablative regime. One of them indicates that it is possible to control the periodicity of the LIPSS by choosing the proper Cr film thickness. A simulation allowed determining the influence of different spatial concentrations of two different Cr oxides, i.e., CrO_2_ and Cr_2_O_3_, along one of the produced ridges. The simulated field spatial distribution demonstrates that changes in the chemistry induced thermally by the laser pulses affect the final intensity pattern imprinted into the material [28]. In another similar system of Cr films, the authors consider different beam scanning speeds to investigate the influence on the transition from HSFL perpendicular to the polarization to LSFL being parallel to it (${\mathrm{LSFL}}^{∥}$), involving the formation of both Cr oxides and the interaction of a surface electromagnetic wave between two dielectric materials, air and glass, in which the Cr film has been deposited [29]. In this case, the proposed model includes the optical properties of both Cr oxides and ratios between them; however, the obtained periodicities are ~1.5 times larger than the ones obtained experimentally. Only by assuming a certain porosity inside the oxide layer, the predicted periodicities are close to the measured values [29]. From all this, it is clear that the incorporation of additional effects, such as superficial oxidation, into the calculations to predict the LSFL periodicity and orientation is not a trivial task.

Therefore, in this paper we present a different approach by numerically simulating a layered system via FDTD calculations of the laser beam intensity inside a superficial laser-generated oxidation layer and at its interface to an underlying substrate of CrN as a model of an oxidation-prone strong absorbing material. Surprisingly, we found instead of the usual ${\mathrm{LSFL}}^{⊥}$ some ${\mathrm{LSFL}}^{∥}$ that were not reported yet for this type of material. These new structures are formed for static and dynamic irradiations of CrN in the ablative regime and could be generated using two different laser systems in two independent laboratories. The irradiated samples were characterized chemically by energy dispersive X-ray analyses (EDX) and µ-Raman spectroscopy, and morphologically by scanning electron microscopy (SEM) and atomic force microscopy (AFM). When comparing the numerical FDTD simulations with the experimental results, we obtain a significant agreement of their characteristics, suggesting that the formation of the laser-induced oxide layer is a key parameter for the formation of the ${\mathrm{LSFL}}^{∥}$ structures. 

## 2. Materials and Methods 

**Figure 1:**
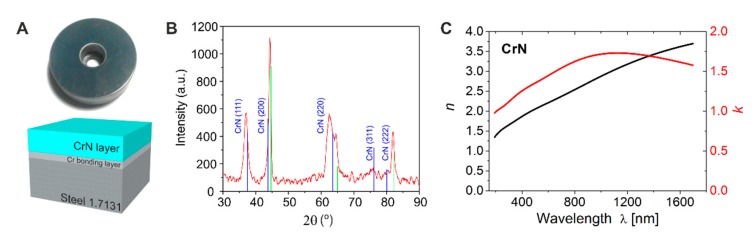
(**A**) Picture of the actual sample with a sketch that illustrates the steel 1.7131 substrate, covered with a 200 nm Cr film for bonding a 2.5 µm thick chromium nitride (CrN) top layer. (**B**) X-ray diffraction (XRD) data of a non-irradiated area of a CrN samples as displayed in (**A**), including peaks corresponding to iron (marked green) and a CrN form known as Carlsbergite (marked blue). (**C**) Plot of the refractive index *n* (left axis) and the extinction coefficient *k* (right axis) of the CrN layer for different wavelengths as measured by ellipsometry. In our calculations we used *n* = 2.439 and *k* = 1.623 for λ = 800 nm.

The samples used for the experiments were provided by Miba Gleitlager GmbH (Laakirchen, Austria) within the frame of the European FET Open research project LiNaBioFluid. They consist of steel disks (16MnCr5, 1.7131) with dimensions of 35 mm diameter and 1 cm height, with a central borehole. The upper flat polished surface was covered via physical vapor deposition (sputtering) with a 2.5 µm thick layer of chromium nitride (CrN) on top of a ~200 nm thin bonding intermediate chromium (Cr) layer, as shown in Figure 1A. The samples were stored in a desiccator and were cleaned in an ultrasonic bath in isopropanol/acetone for 5 min and subsequent drying with pressurized air.

The CrN layer was characterized by X-ray diffraction (XRD). The measurements (XRD 300 TT, Seifert, Ahrensburg, Germany) were performed in Bragg-Brentano geometry using Cu Kα-radiation. Since the element iron from the underlying steel substrate caused a significant X-ray fluorescence, the acquired diffraction curves were corrected by a linear background subtraction. The XRD analysis (Figure 1B) indicates peaks of the Carlsbergite phase of stoichiometric CrN and of the underlying steel substrate. The widths of the CrN peaks and their peak intensities point toward a textured growth of CrN with crystalline grain sizes of several tens up to a few hundreds of nanometers—consistent with the SEM micrograph and AFM data shown in Figure 3A. Optical characterization was performed using a Variable Angle Spectroscopic Ellipsometry (VASE) to determine the optical constants of the CrN layer with a J.A. Woollam M-2000DI ellipsometer (Lincoln, NE, USA) and the WVase32 software (version 3.81) of the same company. The corresponding data of the refractive index *n* and the extinction *k* are presented in Figure 1C in the spectral range of 200 to 1700 nm. From these measurements, the linear absorption coefficient α = 4π*k*/λ and the associated optical penetration depth 1/α can be calculated. At 800 nm (*n* = 2.439, *k* = 1.623) and 1030 nm (*n* = 2.945, *k* = 1.7306) wavelengths, the latter account to 1/α ~39 nm and 47 nm, respectively. The optical penetration depth of the laser radiation in CrN is more than one order of magnitude smaller that the thickness of the CrN layer. Hence, from the optical point of view, the CrN can be considered as bulk material here.

**Figure 2:**
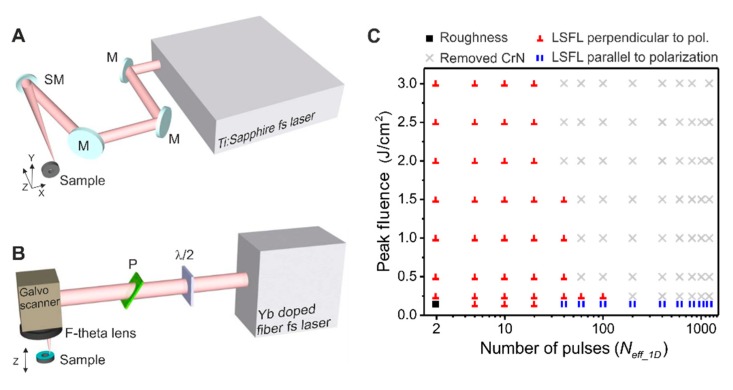
(**A**) Setup of the Ti:Sa laser system (BAM). The beam is directed using several mirrors (M), the polarization is defined by a half-wave plate (λ/2) and is finally focused in a static position on the sample by a spherical dielectric mirror (SM) of 500 mm focal length. The sample is positioned and translated by a motorized *X-Y-Z* translation stage. (**B**) Setup of the Yb-doped fiber laser (CSIC). The beam power is controlled by a combination of a half-wave plate (λ/2) and a thin film polarizer (P). The beam scans the sample by a galvanometric mirror-based scanning head, and it is focused by a 100 mm F-theta lens. The sample is mounted in a *Z*-axis translation stage to control the focusing of the beam. (**C**) Plot that displays the obtained type of surface structures on CrN for a combination of number of effective pulses (*N*_eff_1*D*_) and peak fluences used for the formation of (■) roughness, (**║**) indicates the presence of ${\mathrm{LSFL}}^{∥}$, (**⊥**) indicates the presence of ${\mathrm{LSFL}}^{⊥}$ and (**×**) marks the conditions where the CrN layer has been destroyed.

**Laser irradiation setups.** The irradiation experiments performed involved loosely focused femtosecond laser pulses from two different laser sources: Figure 2A shows a solid state Ti:Sa laser amplifier system (Compact Pro, Femtolasers, Vienna, Austria) operated at 1 kHz repetition rate delivering pulses of 790 nm center wavelength and duration of ~30 fs (full width at half maximum, FWHM).

This setup focused the laser pulses using a spherical broadband dielectric mirror of 500 mm focal length (f/#-number ~130), obtaining a 1/e^2^-beam diameter at the focus of 2*w*_0_ = 130 µm. The sample was mounted normal to the incident focused laser beam and positioned with an *X*-*Y*-*Z* motorized translation stage (VT-80, MICOS, Eschbach, Germany), scanning the sample in the *X*-*Y-*plane and controlling the focus with the *Z*-axis. The second laser system, in Figure 2B, shows an Yb-doped fiber laser (Satsuma HP, Amplitude Systemes, Talence, France) with repetition rates ranging from 1 kHz to 2 MHz emitting pulses of 1030 nm wavelength and 350 fs duration (FWHM). For the experiments presented in this work the repetition rate was fixed at 100 kHz. The pulses were focused through a 100 mm focal length (f/#-number ~20) F-Theta lens coupled with a fast galvanometric-mirror based scanning head (SCANcube 14, Scanlab GmbH, Puchheim, Germany) keeping the beam diameter of 2*w*_0_ = 39 µm on a nominal square area of 70 × 70 mm^2^. The experiments were carried out in the 3 × 1 mm^2^ central region of this working space to avoid any possible modifications due to the beam angle of incidence. In both laser processing systems, the sample was fixed on a holder that allowed the setting of the perpendicularity of the sample surface to the incident laser beam. In addition, during the irradiation, an air suction system was used to extract actively the ablated debris and produced gases in both setups. Figure 2C provides a “morphological map” of the structures that were produced on a CrN sample including LSFL parallel to the laser polarization (^║^) and perpendicular to it (┴). Under a combination of high laser fluences and high number of pulses, the CrN layer is removed from the steel substrate, indicated by the symbol (**×**).

**Irradiation procedure.** For the generation of LIPSS on CrN, we have performed a series of static (spot only) and dynamic (spatially overlapped pulses to form lines) irradiations using different incident peak laser fluences (*ϕ*_0_) and number of effective pulses per spot diameter (*N*_eff_1*D*_). Peak fluences were determined according to the method of Liu [30]. The calculation of *N*_eff_1*D*_ follows the equations presented in [31] that take into account the scanning speed (*v*), laser beam waist radius at the focus (*w*_0_) and the laser pulse repetition rate (*f*) as *N*_eff_1*D*_ = 2*w*_0_*f*/*v*. For the processing of LIPSS-covered areas, the line separation distance (Δ) is considered as an additional parameter. All laser irradiations were performed in air at room temperature.

**Surface characterization.** In order to characterize the sample surface morphology, we use two characterization techniques: SEM (Gemini Supra 40, Carl Zeiss, Oberkochen, Germany) and AFM (Dimension 3100, Digital Instruments, Santa Barbara, CA, USA). The SEM micrographs were acquired in SE-InLens mode at 5 and 10 kV electron acceleration voltage. This imaging mode acquires the so-called SE1 secondary electrons produced mainly at the impact point of the primary electron beam allowing for a high spatial resolution. Two-dimensional Fourier transform (2D-FT) analyses were performed using the free software ImageJ (version 1.52a, National Institutes of Health, Bethesda, MD, USA) to get information on the periodicity of the structures. In order to account the periodicity variation, we performed a radial average around the data cloud of interest in the Fourier space, then we fitted the data into a Gaussian distribution from which we obtained sigma (σ) as the periodicity range displayed in all the 2D-FTs [32]. AFM topographic data of specific samples were acquired in tapping mode using silicon probes with a nominal tip radius of 10 nm. Images contained 512 lines in *X*- and 256 lines in *Y*-direction, allowing a nominal resolution in *Z* of 70 pm (4.7 µm/16 bit in the full *Z-*scale range). The data are displayed as 3D representation of the topography, from which the root-mean-squared (RMS) roughness of the surfaces is obtained and a cross-section topography profile is extracted, parallel to the *X*-axis.

**Chemical and structural characterization of laser-irradiated samples.** Energy dispersive X-ray analyses (EDX) using a Thermo NSS 3.1 detector (Thermo Fisher Scientific, Waltham, MA, USA) were performed on selected samples to obtain the compositional information of the fs-laser irradiated samples. The acceleration voltage in this case was 5 kV to ensure the necessary surface sensitivity. Single point µ-Raman spectra were recorded as five accumulated acquisitions in backscattering geometry to characterize the local material structure and composition in the fs-laser modified regions. The continuous wave 532 nm wavelength laser beam was focused by a 100× microscope objective (numerical aperture NA = 0.85) to a probing spot diameter of ~1 µm, resulting in a power of 0.1 mW at the sample surface.

## 3. Results and Discussion

**Figure 3:**
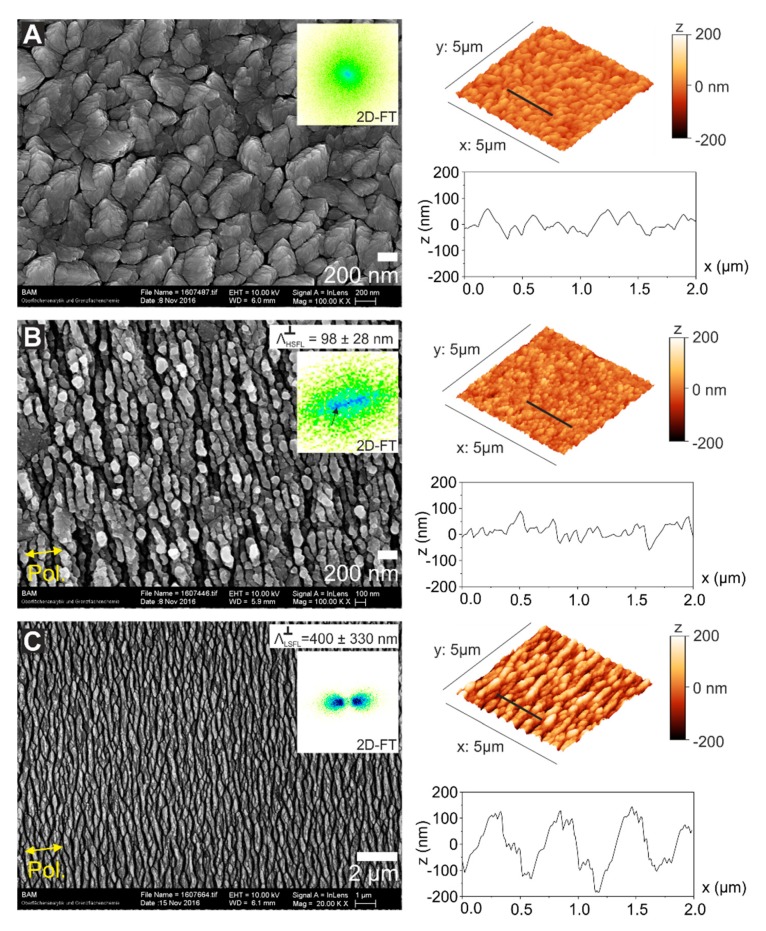
Top-view scanning electron microscopy (SEM) micrographs and 3D reconstructed atomic force microscopy (AFM) data acquired in different areas of the sample with its corresponding two-dimensional Fourier transform to estimate the structures periodicity (Λ). (**A**) Non-irradiated surface showing the randomly oriented CrN grains with no clear periodicity in the Two-dimensional Fourier transform (2D-FT). The AFM data shows a profile taken at the position of the indicated black line, featuring a surface root-mean-squared roughness of $R_{\mathrm{CrN}}$ = 25 nm. (**B**) HSFL produced on an irradiated area at $\phi_{0}\;$= 0.15 J/cm^2^, *N*_eff_1*D*_ = 200 and line separation distance Δ = 50 µm. The periodicity in this case is $\Lambda_{\mathrm{HSFL}}^{⊥}$ = 98 ± 14 nm, the modulation depth is *d*_HSFL_ ~50 nm and the root-mean-squared-roughness is $R_{\mathrm{HSFL}}=$ 30 nm. (**C**) ${\mathrm{LSFL}}^{⊥}$ processed at $\phi_{0}$ = 0.5 J/cm^2^, *N*_eff_1*D*_ = 10 and line separation distance Δ = 50 µm. The periodicity is $\Lambda_{\mathrm{LSFL}}^{⊥}$ = 400 ± 330 nm, the modulation depth *d*_HSFL_ ~200 nm and the root-mean-squared-roughness is $R_{\mathrm{LSFL}}=$ 73 nm. Both structures (Figure 3B,C) were produced with the Ti:Sa system (λ = 790 nm) and the structures orientation is perpendicular to the laser beam polarization, indicated by yellow double arrows.

**Laser irradiations.** The scanning electron micrograph displayed in Figure 3A corresponds to a non-irradiated sample, revealing the CrN grains at the surface. According to the information extracted from the 2D-FT, there is neither a clear periodicity nor orientation of the structures of the pristine surface. From the AFM data, it is possible to obtain the RMS-roughness of $R_{\mathrm{CrN}}$ = 25 nm. The fs-laser irradiations follow the procedure described in the “Materials and Methods” section. There are two different irradiation strategies: areas processed by dynamically scanning the sample surface with the laser beam or static irradiation of fixed spot locations.

The first set of experiments contained dynamic irradiations to process 2 × 1 mm^2^ areas using a Ti:Sa laser system ($\phi_{0}\;$= 0.15 J/cm^2^, *N*_eff_1*D*_ = 200, Δ = 50 µm). These parameters are defined in “Materials and Methods” section. At this fluence, the final morphology of the laser-induced structures corresponds to HSFL oriented perpendicular to the laser beam polarization, as displayed in Figure 3B. The periodicity obtained in this case from the 2D-FT of the SEM micrograph is $\Lambda_{\mathrm{HSFL}}^{⊥}$ = 98 ± 14 nm, and the RMS roughness $R_{\mathrm{HSFL}}=$ 30 nm. From the AFM cross-sectional profile, it can be seen that the HSFL are superimposed to the roughness of the initial topography of the CrN surface. Note that the measurement of the RMS roughness takes into account the whole surface data including both, the CrN grain roughness with the superimposed laser-generated HSFL, the topography of which can be better observed in the profile of Figure 3B.

Another laser-processed area is produced using an approx. three-times larger laser fluence allowing the fabrication of LSFL perpendicular to the laser polarization ($\phi_{0}$ = 0.5 J/cm^2^, *N*_eff_1*D*_ = 10, Δ = 50 µm). The corresponding surface morphology is shown in Figure 3C, including an SEM micrograph and AFM data. The periodicity range of the structures extracted from the 2D-FT of the SEM micrograph is $\Lambda_{\mathrm{LSFL}}^{⊥}$ = 400 ± 330 nm. The large period variation of about 83% is partly attributed to the effects of period splitting via localized electromagnetic field-enhancement effects [33]. Moreover, the consequences of laser scanning must be considered, where the ${\mathrm{LSFL}}^{⊥}$ generated at the high fluence part of the Gaussian laser beam are subsequently “overwritten” by HSFL formed by the following low-fluence wing of the spatial beam profile. From the AFM cross-section, it is possible to estimate a periodicity of the footprint of the bigger $\Lambda_{\mathrm{LSFL}}^{⊥}$ structures of ~600 nm and an RMS roughness $R_{\mathrm{LSFL}}$ = 73 nm, in good agreement with the work of Yasumaru et al. studying the characteristics of fs-laser generated LIPSS on CrN [25].

**Figure 4:**
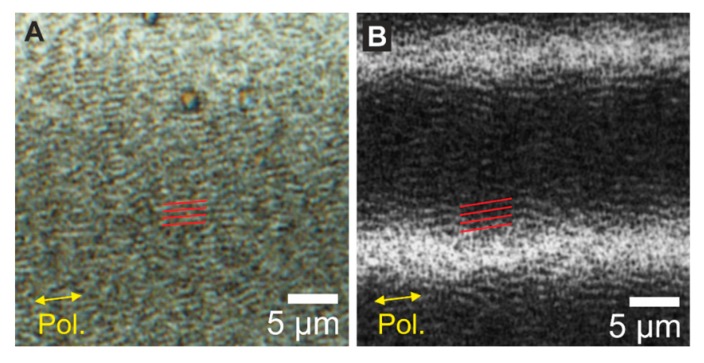
Morphological characterization via top-view optical microscopy (OM) of the samples irradiated with (**A**) Ti:Sa laser system (λ = 790 nm), using the same parameters of Figure 3B, featuring the structures parallel to the laser polarization, highlighted with a set of four parallel red lines, and (**B**) Yb-doped fiber laser system (λ = 1030 nm, $\phi_{0}\;$= 0.15 J/cm^2^, *N*_eff_1D_ = 1000), featuring ${\mathrm{LSFL}}^{∥}$ structures parallel to the laser polarization highlighted with a set of four parallel red lines. In both cases, the yellow double arrows indicate the polarization direction.

Surprisingly, when observing the irradiated areas for both laser systems under a regular optical microscope, as shown in Figure 4, it is possible to detect another type of regular LSFL (${\mathrm{LSFL}}^{∥}$), structures (indicated by the sets of red parallel lines in Figure 4) being parallel to the polarization (as indicated by the yellow double-arrows.) Importantly, from the AFM data shown in Figure 3B the only detected structures at the sample surface are HSFL perpendicular to the polarization, which clearly confirms that the ${\mathrm{LSFL}}^{∥}$ are not located at the surface but constitute in a sub-surface layer. 

More in detail, Figure 4A shows the morphologies detected in areas processed with the Ti:Sa laser system, where the topography corresponds to ${\mathrm{LSFL}}^{∥}$ with periodicities of $\Lambda_{\mathrm{LSFL}}^{∥}$ = 820 ± 30 nm. Since the topography corresponds to the same region shown in Figure 3B, it can be assumed that HSFL are present at the surface despite that is not possible to resolve them via optical microscopy. The ${\mathrm{LSFL}}^{∥}$ fabricated with the Yb-doped fiber laser system ($\phi_{0}\;$= 0.15 J/cm^2^, *N*_eff_1*D*_ = 1000, Δ = 20 µm) are shown in Figure 4B, presenting a periodicity of $\Lambda_{\mathrm{LSFL}}^{∥}$ = 1.26 ± 0.08 µm.

**Figure 5:**
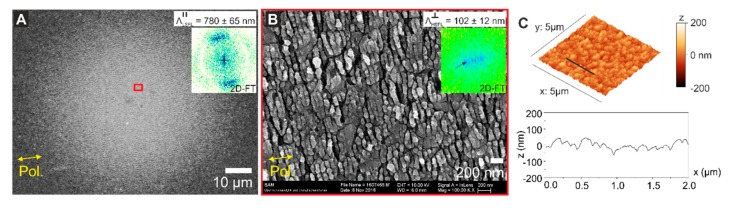
(**A**). Top-view SEM micrograph of a spot made under the same irradiation conditions of the area shown in Figure 3B and Figure 4A ($\phi_{0}$ = 0.15 J/cm^2^ and *N* = 200), acquired in InLens mode at 5 kV. It is also possible to detect the formation LSFL parallel to the beam polarization with periodicity $\Lambda_{\mathrm{LSFL}}^{∥}$ = 780 ± 65 nm. (**B**) SEM micrograph of the red square inset in (**A**). In this case, the magnification factor is 100.000× and the acceleration voltage was 10 kV, showing the presence of HSFL perpendicular to the polarization with a periodicity $\Lambda_{\mathrm{HSFL}}^{⊥}$ = 102 ± 12 nm. (**C**) AFM 3D-data including a profile taken on the indicated black line showing an RMS roughness $R_{\mathrm{HSFL}}=\;$32 nm.

In order to discard possible effects due to the laser beam scanning on the${\;\mathrm{LSFL}}^{∥}$, we performed an additional static (*N*-on-1) irradiation experiment in a single spot with the Ti:Sa laser system under the same experimental conditions as in Figure 3B and Figure 4A ($\phi_{0}\;$= 0.15 J/cm^2^, *N* = 200). The corresponding SEM micrograph is included in Figure 5A, taken with an acceleration voltage of 5 kV. Similarly, LSFL structures parallel to the laser beam polarization are present with a periodicity of $\Lambda_{\mathrm{LSFL}}^{∥}$ = 780 ± 65 nm, which confirms that the phenomenon does not depend on the scanning overlap of the laser pulses. A second micrograph is acquired at higher magnification and acceleration voltage of 10 kV (Figure 5B), showing HSFL similar to those of Figure 3B, with periodicities of $\Lambda_{\mathrm{HSFL}}^{⊥}$ = 102 ± 12 nm. In order to confirm that the ${\mathrm{LSFL}}^{∥}$ are not located at the sample surface, AFM data displaying the topography is included in Figure 5C, where again only HSFL structures superimposed to the CrN grains can be observed.

**Figure 6:**
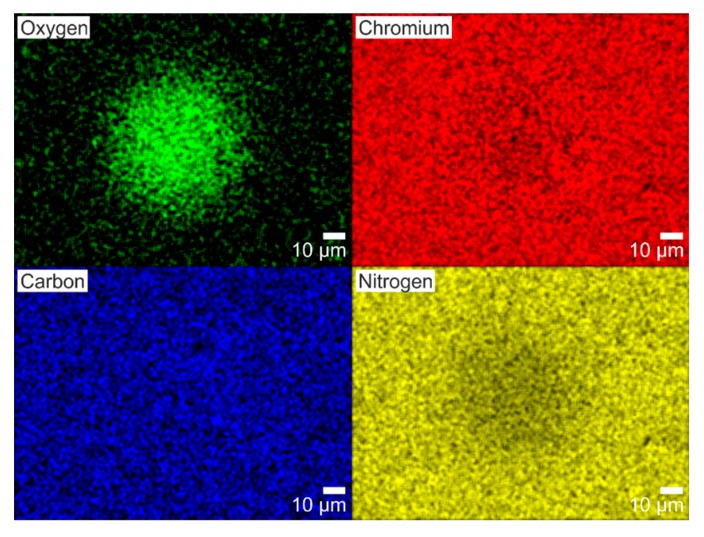
EDX analysis of the fs-laser irradiated spot shown in Figure 5A, where the concentrations of oxygen, chromium, carbon and nitrogen are displayed in false colors. Brighter colors indicate larger signals. Note that the spatial distribution of oxygen is consistent with the irradiated spot shown in Figure 5A.

**Chemical characterization.** Additional analyses were performed for the material modification accompanying the formation of the ${\mathrm{LSFL}}^{∥}$ structures. A first chemical characterization of the surface was conducted by EDX analysis on the spot shown in Figure 5A using an acceleration voltage of 5 kV to ensure the necessary surface sensitivity. The results are compiled in Figure 6, where the spatial distribution of oxygen (O), chromium (Cr), carbon (C) and nitrogen (N) are encoded in false colors. The EDX elemental map of O confirms an increased concentration of oxygen in the irradiated area keeping the same size and shape of the irradiated spot. Contrary to chromium and carbon, where the concentration is practically the same in the irradiated area and its surroundings, the nitrogen concentration slightly decreases in the irradiated area. This indicates that the oxygen is located close to the sample surface and is, thus, reducing the signal of the nitrogen from underneath due to the formation of a superficial oxidation layer on the CrN. Those results further support the idea that the formation of the ${\mathrm{LSFL}}^{∥}$ structures may take place at the interface between the laser-induced oxidation layer and the underlying CrN material, in line with the findings obtained from the SEM/AFM characterizations.

**Figure 7:**
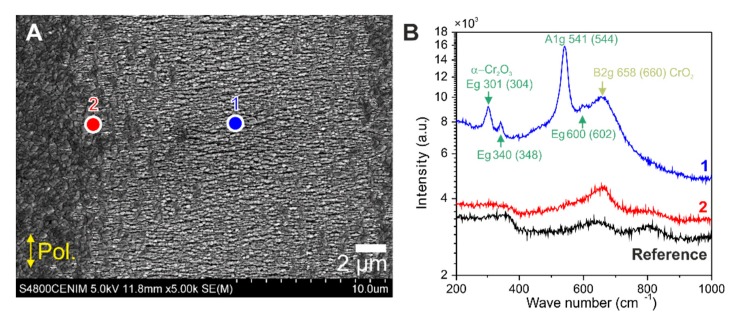
(**A**) Top-view SEM micrograph taken at 5 kV of an irradiated line on the CrN surface using the Yb-doped fiber laser system. The whitish regions indicate that the laser-processing generates LSFL^║^ visible in the borders of the line (red dot) parallel to the beam polarization and HSFL in the center (blue dot) perpendicular to the laser beam polarization (indicated by the yellow double-arrows). Irradiation conditions are the same as those of Figure 4B. (**B**) µ-Raman spectra acquired at the positions indicated by the red and blue dots in (**A**) with an additional spectrum that corresponds to a non-irradiated area (black line—dot not shown in (**A**) for comparison. Note the logarithmic scale for the intensity axis.

Complementary structural information was gained from µ-Raman spectroscopy. Figure 7 presents results of the structures produced on a line track produced with the Yb-fiber laser system under the same conditions as the one presented in Figure 4B. In this case, the SEM micrograph of Figure 7A was taken using 5 kV electron acceleration voltage. The LSFL structures are oriented parallel to the polarization direction, indicated by the yellow double-arrows. However, the morphology is dominated by HSFL structures perpendicular to the polarization, presenting a periodicity of $\Lambda_{\mathrm{HSFL}}^{⊥}$ = 213 ± 35 nm. The value is larger than the one corresponding to Figure 3B mainly due to the increased laser wavelength (λ = 1030 nm) and the somewhat different irradiation conditions used in this case. 

In order to get detailed information of the composition of the oxide layer, µ-Raman spectra were acquired on different positions across the fs-laser processed line track. For experimental details of the µ-Raman characterization, see the “Materials and Methods” section. The µ-Raman spectra plotted in Figure 7B were recorded on a non-irradiated location of the sample (black line), at the border of the fs-laser track (red line) and at its center (blue line) in the two positions marked in Figure 7A. When comparing to the spectra from the non-irradiated area, both the border of the laser track and its center present an increased Raman signal around 660 cm^−1^. According to [34], it corresponds to the B_2g_ mode of CrO_2_. The Raman spectrum from the border of the track does not present any other identifiable peaks attributed to oxides. In the case of the center of the laser track (blue line), the Raman spectrum shows the presence of several pronounced peaks on a broad background that confirm the presence of the corundum structure of Cr_2_O_3_ with modes A_1g_ (541 cm^−1^) and E_g_ (301, 340 and 600 cm^−1^) [35,36].

**FDTD Simulations.** In order to check the hypothesis whether the ${\mathrm{LSFL}}^{∥}$ may be formed at the chromium oxide/chromium nitride interface, and more specifically, to identify the role of the oxide layer and its thickness, we have employed the finite-difference time-domain (FDTD) method to calculate the spatial intensity distribution induced by the fs-laser beam at the interface between the CrN layer and the covering oxide layer. It was assumed that the oxide layer is composed mainly of Cr_2_O_3_ to simplify the calculations to a single material for the oxide layer. For these simulations, we numerically solved the Maxwell’s Equations (1) and (2), where $\vec{H}$ is the magnetizing field, $\vec{E}$ the electric field, $\epsilon_{0}$ the electric permittivity of free space and $\mu_{0}$ permeability of free space,

(1) $$\vec{\nabla }\times \vec{H}=\epsilon_{0}\epsilon_{r}\frac{\partial \vec{E}}{\partial t}+\sigma \vec{E}$$

(2) $$\vec{\nabla }\times \vec{E}=-\mu_{0}\frac{\partial \vec{H}}{\partial t}$$

considering the relative permittivity $\epsilon_{r}$ defined as:

(3) ϵr=Re(n˜)2−Im(n˜)2

in addition, the conductivity σ as:

(4) σ=2ϵ0ωRe(n˜) Im(n˜)

where $\tilde{n}=n+ik$ is the complex refractive index of the medium and ω is the angular frequency of the laser radiation (ω = 2.35 × 10^15^ s^−1^ for λ = 800 nm irradiation wavelength). The optical constants n and k for the CrN layer were measured experimentally for the substrate system using ellipsometry measurements of a non-irradiated sample, see Figure 1C in the “Materials and Methods” section: *n* = 2.439 and *k* = 1.623 for λ = 800 nm. For the oxidized layer of Cr_2_O_3_, *n* = 2 and *k* = 0 were found in [37], and for vacuum *n* = 1 and *k* = 0.

The laser beam considered had Gaussian temporal and spatial distributions. At the surface of the sample it is considered as a plane wave, polarized along the *X*-axis. The temporal envelope of the electric field at the sample surface is described by:

(5) $$\vec{E}\left(z=0,t\right)=E_{0}\exp\left[-{\left(\frac{t}{\tau }\right)}^{2}\right]\sin\left(\omega t\right){\hat{e}}_{x}$$

**Figure 8:**
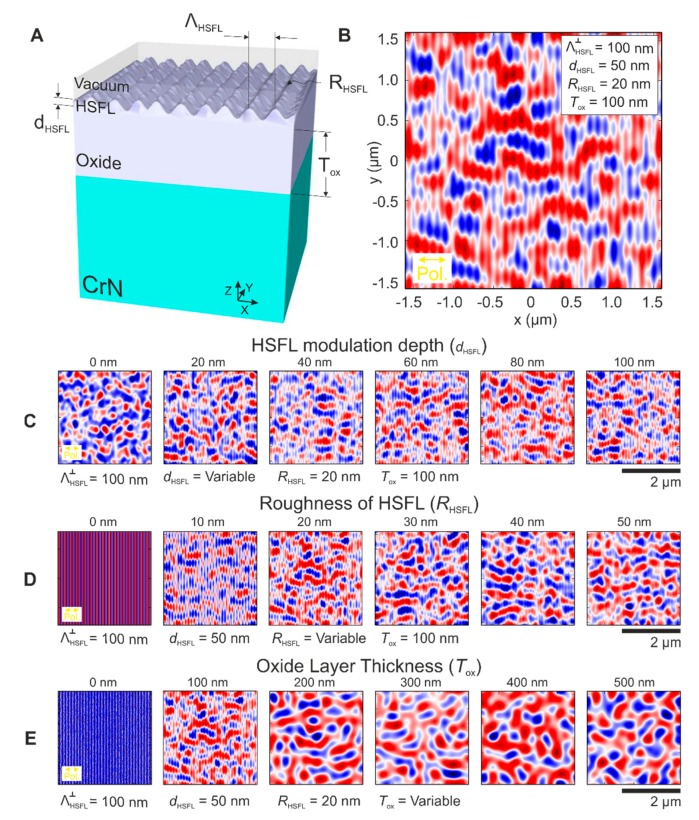
(**A**) 3D-representation (not to scale) of the simulated domain with dimensions in *X* and *Y* of 3.2 µm and variable *Z* including the different layers, visualizing the relevant parameters studied in the finite-difference time-domain (FDTD) simulations. (**B**) Intensity pattern obtained at the interface of the oxide layer and the CrN sample for a sample with HSFL periodicity $\Lambda_{\mathrm{HSFL}}^{⊥}\;$= 100 nm, HSFL modulation depth *d*_HSFL_ = 50 nm, roughness of the HSFL $R_{\mathrm{HSFL}}\;$= 20 nm, and oxide layer thickness *T*_ox_ = 100 nm. Subsequent simulations take into account the same conditions as in (**B**) changing in (**C**) *d*_HSFL_, from 0 to 100 nm, (**D**) $R_{\mathrm{HSFL}}$ from 0 to 50 nm and in (**E**) *T*_ox_ from 0 to 500 nm. The laser beam (λ = 800 nm) polarization direction is horizontal. The colors of the intensity plots are encoded in a false color scheme where red indicated deviations above and blue below the average intensity value of the image, represented in white.

The 3D-geometry used to compute the results is included in Figure 8A. The simulation domain sizes were fixed at 3.2 µm in *X*- and *Y*-directions. The *Z*-dimension was adjusted in each case depending on the thickness of each simulated element. The 3D-domain was subdivided into 3D-space cells which sizes were 5 nm for *X* and *Y* and 2 nm for *Z*. From bottom to top, there is a layer of CrN that starts at *Z* = −∞ and finishes at the interface with the oxide layer (*Z* = 0). In our simulation, the real CrN layer thickness of 2.5 µm is thick enough to be considered optically as an infinite layer (see the values of the optical penetration depth of some tens of nanometers provided in the “Materials and Methods” section). Therefore, the steel substrate and the chromium-bonding layer were excluded for the simulations since they do not have any relevant optical contribution. On top of the CrN layer, there is an oxide film that has a specific thickness $T_{ox}$ ranging from 0 to 500 nm. On top of the oxide layer, and made up of the same oxidized materials, lay the grating-like HSFL structures with periodicity $\Lambda_{\mathrm{HSFL}}$ = 100 nm for all the simulations. This value is consistent with the HSFL periodicity found via 2D-FT of Figure 3B and Figure 5B, $\Lambda_{\mathrm{HSFL}}^{⊥}$ = 98 ± 14 nm and 102 ± 12 nm, respectively. At the sample surface, there are two variables to consider: the modulation depth of the HSFL $d_{\mathrm{HSFL}}$ ranging from 0 to 100 nm, and a non-organized roughness $R_{\mathrm{HSFL}}$ ranging from 0 to 50 nm. The rest of the domain is always large enough to contain at least 10 nm of vacuum above the HSFL region. The simulations ran from *t* = −2τ to *t* = 2τ, with τ = 25 fs (FWHM = 2τln2 ≈ 35 fs pulse duration) using time steps of 5.516 attoseconds. We computed the time-averaged intensity in arbitrary units during the whole interaction, following:

(6) $$I=\int_{-2\tau \;}^{2\tau \;}\left(E_{x}^{2}+E_{y}^{2}+E_{z}^{2}\right)dt$$

All simulated intensity distributions that were evaluated at the interface between the oxide layer and the CrN sample are compiled in Figure 8. Figure 8B presents a simulation of the intensity in a sample modelled with a HSFL modulation depth *d*_HSFL_ = 100 nm, an oxide layer of thickness *T*_ox_ = 100 nm and an additional non-organized roughness $R_{\mathrm{HSFL}\;}$= 20 nm. Under these conditions, that are very close to the real HSFL structures observed in the experiments of Figure 3 and Figure 5, it is already possible to recognize the signature of the ${\mathrm{LSFL}}^{∥}$ with period $\Lambda_{\mathrm{LSFL}}^{∥}$= 580 ± 98 nm. Some remnants of the interaction between the laser and the HSFL grating at the surface are still present and manifest as lines oriented along the *Y*-axis with a periodicity of 100 nm in the *X*-direction. The other simulations shown in Figure 8 address systematic variations of the HSFL modulation depth $d_{\mathrm{HSFL}}$ (Figure 8C), the roughness parameter *R*_HSFL_ (Figure 8D), and the oxide layer thickness *T*_ox_ (Figure 8E), respectively. In order to observe its influence on the formation of the intensity pattern responsible of the ${\mathrm{LSFL}}^{∥}$ formation, the FDTD simulations were performed changing one parameter each time, while keeping the others constant, as it is indicated in each set of sub-figures.

The simulations obtained upon changing the modulation depth ($d_{\mathrm{HSFL}}$) of the HSFL gratings formed on the oxide layer, are shown in Figure 8C. Without HSFL structures on the oxide layer ($d_{\mathrm{HSFL}}$ = 0 nm), the intensity distribution corresponds to a non-organized pattern, only affected by the initial roughness present at the oxide surface set to $R_{\mathrm{HSFL}}$ = 20 nm here. For metals it has been demonstrated that the roughness at the surface is an important parameter that may influence the final periodicity of the LIPSS [8,17]. However, this is only valid when the structures are produced at the first irradiated interface (the surface), in our case, the oxide layer, and not at the interface between two different materials. For all the other modulation depths shown $(d_{\mathrm{HSFL}}>\;$0 nm), there is a two-fold modulation in the calculated intensity patterns at the oxide/nitride interface: in the *Y-*axis direction, the HSFL remnants are still visible and are practically constant for all the studied $d_{\mathrm{HSFL}}$ values. In the *X*-axis direction, there is certain modulation in the form of LSFL parallel to the laser polarization, significantly more pronounced when $d_{\mathrm{HSFL}}$ ≳ 40 nm. This result is consistent with the experimentally measured modulation depths for the HSFL shown in Figure 3B and Figure 5C (AFM data), having values of $d_{\mathrm{HSFL}}$ ~50 nm for both cases.

The second parameter varied in the simulation was the non-organized roughness superimposed to the HSFL covered oxide layer, $R_{\mathrm{HSFL}}$, as shown in Figure 8D. Note that this parameter is different from the root-mean-squared roughness obtained from the AFM data in Figure 3B and Figure 5C. When $R_{\mathrm{HSFL}}$ = 0 nm, the sample surface corresponds to a smooth HSFL grating. Since the symmetry is not broken due to the absence of any surface defects, it is expected that the intensity along the *Y*-axis is not modulated in the corresponding FDTD simulations. As $R_{\mathrm{HSFL}}$ increases, the relative signature of the HSFL is drastically reduced and the intensity modulation parallel to the laser polarization (*X*-axis) featuring the ${\mathrm{LSFL}}^{∥}$ characteristics becomes evident, reaching average values of $\Lambda_{\mathrm{LSFL}}^{∥}$ = 650 ± 50 nm when $R_{\mathrm{HSFL}}$ = 40 nm, in reasonable agreement to the experimental periodicities found in Figure 5A for the interfacial LSFL $\Lambda_{\mathrm{LSFL}}^{∥}$ = 780 ± 65 nm.

The final parameter varied is the oxide layer thickness, *T*_ox_, as shown in Figure 8E. When *T*_ox_ = 0 nm, the incident laser beam has propagated only through vacuum, and no dispersive or reflecting interface elements modify the wave front upon propagation. Hence, apart from the influence of the non-organized surface roughness and remnants of the HSFL grating, there is no significant modulation on the laser beam intensity observed here. The periodicity that can be seen in this case, corresponds to the presence of the HSFL that are present at the surface of the CrN layer. When *T*_ox_ = 100 nm, the intensity is already modulated in the *Y*-direction while still exhibiting the signature of the HSFL in the *X*-direction. For even thicker oxide layers, the regularity of the intensity pattern is reduced and even the contribution from the HSFL disappears due to propagation effects in the oxide and the superposition of the incident waves with that partially reflected from the CrN interface.

**Figure 9:**
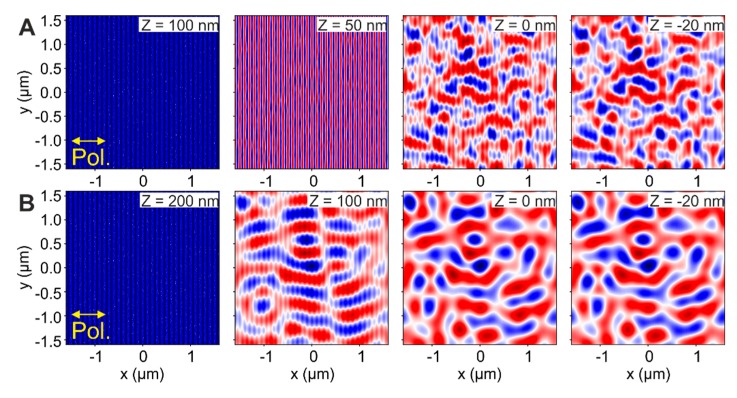
**FDTD-**intensity distribution at different *Z*-positions along the beam axis for two different layered systems with HSFL modulation depth *d*_HSFL_ = 50 nm and. In (**A**) the oxide layer thickness is *T*_ox_ = 100 nm and in (**B**) *T*_ox_ = 200 nm. The interface between the oxide layer and the CrN is located at *Z* = 0 nm. The plots at *Z* = −20 nm in both cases correspond to intensities calculated 20 nm inside the CrN from the oxide-nitride interface. The laser beam (λ = 800 nm) polarization direction is horizontal. The colors of the intensity plots are encoded in a false color scheme where red indicated deviations above and blue below the average intensity value of the image, represented in white.

More in detail, in order to see how the beam is propagating inside this layered system, Figure 9 provides intensity distributions at different positions *Z* along the beam propagation axis. Each plot show insets with the corresponding *Z*-positions at which the intensity distribution is calculated. The position *Z* = 0 nm correspond to the interface between the oxide layer and the CrN sample underneath. In general, positive *Z*-positions indicate that the intensity distribution is located inside the oxide layer (or above it) and negative *Z*-positions correspond to intensities inside the CrN material. The results for a layered system with a modulation depth of *d*_HSFL_ = 50 nm, roughness *R*_HSFL_ = 20 nm and oxide layer thickness *T*_ox_ = 100 nm and 200 nm are compared in Figure 9A and Figure 9B, respectively.

In Figure 9A (top row), where the oxide layer thickness is 100 nm, the intensity distribution is practically unchanged at *Z* = 100 nm, corresponding to the position at the base of the HSFL structures. In the middle of the oxide layer (*Z* = 50 nm) the signature of the HSFL is strongly visible and at this small propagation distance along the *Z*-axis, the beam pattern symmetry is not completely broken yet along the *Y*-axis. At the interface between the oxide layer and the CrN sample (*Z* = 0 nm) the signature of the HSFL is still present but slightly reduced, presenting a clear variation along the *Y*-axis with the signature of LSFL parallel to the laser polarization (LSFL^║^). This pattern is maintained inside the CrN sample reducing significantly the signs of the HSFL, as it is shown in the plot at *Z* = −20 nm.

In Figure 9B (bottom row) a similar system with a just thicker oxide layer of *T*_ox_ = 200 nm is analyzed. The intensity distribution at *Z* = 200 nm is calculated at the base of the HSFL where the intensity distribution remains unchanged (similarly as the one at Z = 100 nm in Figure 9A). In the middle of the oxide layer (*Z* = 100 nm), the modulation of the intensity already shows the very pronounced imprint of LSFL^║^ with a contribution from the HSFL top grating. At the oxide/nitride interface *Z* = 0 nm, the signature of the HSFL is vanished presenting a smooth intensity distribution that corresponds to the LSFL^║^. This distribution is preserved inside the CrN layer (*Z* = −20 nm). From these results, it is possible to conclude that for a layered system with HSFL of 100 nm periodicity, HSFL modulation depth *d*_HSFL_ = 50 nm, roughness $R_{\mathrm{HSFL}}$= 20 nm, (all in good agreement with the experimental parameters found in Figure 3B and Figure 5B), the oxide layer thickness value should be considered between 100 < *T*_ox_ < 200 nm to produce effectively LSFL^║^.

**Figure 10:**
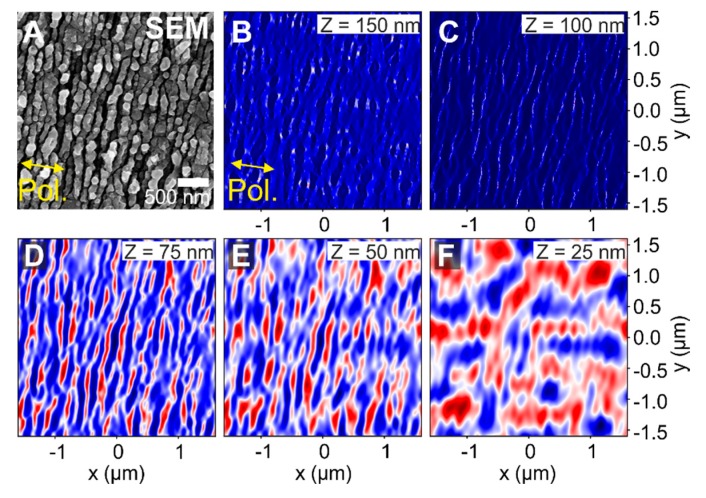
FDTD simulation of a layered system that includes periodic HSFL $\Lambda_{\mathrm{HSFL}\;}^{⊥}$ = 100 nm given by the real structures shown in the SEM micrograph on the top left (**A**), HSFL modulation depth *d*_HSFL_ = 75 nm, no added roughness ($R_{\mathrm{HSFL}}\;$= 0 nm) and oxide layer thickness *T*_ox_ = 100 nm. The intra oxide film position varies between *Z* = 150 nm (**B**), *Z* = 100 nm (**C**), *Z* = 75 nm (**D**), *Z* = 50 nm (**E**), and *Z* = 25 nm (**F**). The laser beam (λ = 800 nm) polarization direction is horizontal. The colors of the intensity plots are encoded in a false color scheme where red indicated deviations above and blue below the average intensity value of the image, represented in white.

For addressing a less idealized simulation scenario, Figure 10 includes the results of a similar layered system considering a more realistic topography for the HSFL structures. The parameters for this simulation are HSFL real periodicity $\Lambda_{\mathrm{HSFL}}^{⊥}\;$= 100 nm, an artificial HSFL modulation depth *d*_HSFL_ = 75 nm based on a false color grayscale image of the SEM micrograph shown in Figure 10A, no additional roughness ($R_{\mathrm{HSFL}\;}$= 0 nm) and an oxide layer thickness of *T*_ox_ = 100 nm.

In this case, the intensity distribution was acquired at different positions only inside the oxide layer (at positive *Z*-positions). The top surface (not shown) is located at *Z* = 175 nm, (*T*_ox_ = 100 nm + *d*_HSFL_ = 75 nm). At *Z* = 150 nm (Figure 10B), it is possible to recognize the morphology shown in the SEM micrograph. The intensity distribution remains essentially unchanged due to the short distance that the beam has propagated inside the oxide layer, similarly as in the case of the HSFL structures (*Z* = 100 nm, Figure 10C). From there, once the beam propagates 25 nm more along the beam axis (*Z* = 75 nm, Figure 10D), the signature of the HSFL is strong and slowly fades away as the beam propagates towards the oxide/nitride interface. At Z = 25 nm (Figure 10F), the LSFL^║^ characteristics with an orientation parallel to the laser beam polarization and larger periods are evident. Remarkably, considering the real topography, the intensity distributions along different positions along the *Z*-axis present a good agreement with the more idealized simulations presented in Figure 8 and Figure 9. This ultimately confirms the relevance of the oxidation at the surface for the fabrication of such buried (interfacial) LSFL structures parallel to the laser beam polarization on oxidation prone materials.

In view of their orientation parallel to the laser beam polarization, their spatial periods of the order of λ/*n*, and their sub-surface presence in a transparent material, it is very likely that the physical origin of the simulated intra-film and interfacial intensity patterns carrying the LSFL^║^ characteristics here lies in the type-d (“dissident”) electromagnetic field structures identified earlier in FDTD simulations [16,18,20,38]. These intensity patterns are formed via the far-field interference between the electromagnetic field scattered at the rough surface and the laser beam refracted into the film and propagating in the sub-surface dielectric material [18,38]. A visualization of the electromagnetic fields emitted from single and multiple type-d scattering centers on dielectric surfaces predominantly in the direction perpendicular to the polarization can be found in [18,39]. Note that the type-m (“mixed”) features observed in FDTD (associated with LSFL parallel to the polarization and periods close to the wavelength λ) can be ruled out here as origin of the LSFL^║^ since the necessary condition of n=Re(n˜)≈ Im(n˜)=k [20] on the optical properties of the oxide material is not fulfilled in our case here (*n* = 2, *k* = 0 [37]). Interestingly, the spatial periods of the sub-surface LSFL^║^ patterns simulated by FDTD rather lie between λ and λ/*n* as it was previously reported in [21]. The deviation between the experimentally observed and numerically simulated periods $\Lambda_{\mathrm{LSFL}}^{∥}$ may arise from the fact that in the experiments the laser-generated oxide layer may not a have a sharp interface to the bulk but may exhibit a graded composition [26] between the sample surface and the CrN bulk material. The latter would be associated then with optical constants varying at depth. Moreover, high-intensity laser-induced transient changes of the refractive index *n* may result in somewhat increased spatial periods (for details refer to the basic Drude model presented in [19]).

## 4. Conclusions

**Figure 11:**
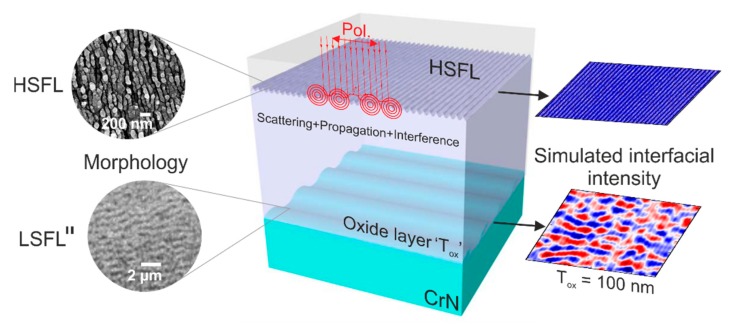
Scheme of the interfacial ${\mathrm{LSFL}}^{∥}$ formation, featuring scattering, propagation and interference effects.

From the presented results, four conclusions can be drawn: (i) it can be clearly observed that the oxide layer is an important parameter that influences strongly the formation of a regular intensity pattern at the interface to the underlying material that presumably leads to the formation of the LSFL structures parallel to the laser polarization. (ii) in order to allow the formation of a regular intensity pattern that can be imprinted at the interface between the superficial oxide layer and the underlying material (CrN), the thickness of the oxide layer should be in the order of ~100 nm, as it has been also suggested in [28]. (iii) the presence of superficial sub-wavelength HSFL on the oxide layer is essentially required to form the near-wavelength ${\mathrm{LSFL}}^{∥}$. (iv) the intensity pattern carrying the spatial ${\mathrm{LSFL}}^{∥}$ characteristics is formed via the joint action of electromagnetic scattering, propagation, and interference effects and does not require the presence of a partially metallic oxide layer (via laser-excited conduction band electrons) or that of nano-plasmas scattering from localized defects [18], as visualized in the summarizing scheme presented in Figure 11. The findings suggest that these effects can be present on strong absorbing materials that are prone to oxide formation.
